# Researching childhood vaccine hesitancy in the wake of COVID-19

**DOI:** 10.1016/j.jvacx.2024.100450

**Published:** 2024-01-26

**Authors:** Alistair Anderson, Tom Douglass, Pru Hobson-West

**Affiliations:** a School of Sociology and Social Policy, University of Nottingham, NottinghamNG7 2RD, United Kingdom; b Department of Social Work and Social Care, University of Birmingham, BirminghamB15 2TT, United Kingdom; c School of Veterinary Medicine and Science, University of Nottingham, LeicestershireLE12 5RD, United Kingdom

**Keywords:** Qualitative methods, Vaccine hesitancy, COVID-19, Childhood vaccination, Immunisation

## Abstract

The COVID-19 pandemic has had a seismic effect on public healthcare, vaccine production, and on society. However, the pandemic has also had a methodological impact on social researchers, including those seeking to better understand vaccine hesitancy in relation to childhood vaccines. In this short communication, we describe the challenging experience of recruiting and conducting qualitative interviews with UK healthcare professionals and vaccine hesitant parents in early 2022. We also explore the way in which the context of COVID influenced our data analysis. Finally, we make recommendations for how researchers, including those using qualitative or quantitative methods, might learn from our experiences, as the complex and delicate relationship between society and vaccines continues to evolve in the wake of the pandemic.

## Introduction

The COVID-19 pandemic led to new vaccine roll-outs, and sometimes heated public discourse around vaccination. The aim of this short research communication is to share critical reflections on our experience of conducting qualitative social research into childhood vaccine hesitancy whilst navigating the COVID-19 context, and to make recommendations for future research.

Previous research suggests that public attitudes to, and engagements with, vaccines and vaccination are complex, and not simply the result of information deficits or non-biomedical approaches to health and healthcare [6]. For example, caution around vaccination may be underlaid by historical relationships between disadvantaged ethnic groups and medical authorities which engender distrust [17], personal and family histories including side-effects, or concerns about the very ways in which vaccination risk is constructed [9]. Hesitancy can also be expressed in relation to specific vaccines, for example in terms of theories linking MMR vaccination and the development of autism symptoms in children [19], and concerns over the HPV vaccination and adolescent sexual behaviour [15]. These examples illustrate the WHO Working Group on Vaccine Hesitancy’s argument that hesitancy is ‘complex and context specific, varying across time, place and vaccines’ ([12] p4163).

A 3-year project on vaccine hesitancy across 7 European Countries was funded by the European Union beginning in 2021, focusing on childhood vaccination and the relationship between healthcare professionals and hesitant parents in specific regions. For this project, hesitancy was understood as a broad social phenomenon, including concern, doubt, or discussion about vaccination. In the UK, the focus was the East Midlands region and the NHS (2022) recommended childhood schedule. The East Midlands – located in the centre of England – has relatively higher levels of negative perception regarding vaccines [1].

In 2022, the authors carried out qualitative interviews with parents and healthcare professionals involved with childhood vaccination. The latter included interviews with health visitors (community public health nurses), general practice nurses, general practitioners, and paediatricians. The original target was 30 interviews in each group, starting in January 2022, allowing 8 months for recruitment. As we discuss, both interview recruitment and interview analysis were challenged by features of COVID-19 context. By identifying these difficulties in each phase, we are able to draw recommendations for future studies into vaccine hesitancy.

## Interviewing vaccine hesitant parents: Recruitment and analytical challenges

Prior to the onset of COVID-19, research has highlighted sampling and parental recruitment as challenging. However, this has arguably been exacerbated by the COVID-19 pandemic [8]. More specifically, the UK team experienced two particular problems that delayed and limited the pace and success of recruitment and negatively impacted the dataset relative to the project objectives.

First, in order to recruit vaccine hesitant parents, the authors used multiple approaches to access this heterogeneous, hard-to-reach population [8]. Initial attempts included the use of social media, personal and professional networks, contacts with complementary and alternative medicine providers, and leaders representing communities that tend to have lower vaccine coverage. Parents’ willingness to consent to participation in an interview about childhood vaccines was found to be reduced by the pandemic context, as some potential interviewees perceived that critical or sceptical views about vaccination in general were under increased attack. This was similar for gatekeepers, as exemplified in an email exchange with a ‘vaccine-critical group’ [9]regarding potential interview participation. The group’s founder voiced concerns about ‘hostility’ and name-calling (such as references to being ‘anti-vax’) coming from the mainstream media, politicians, and healthcare professionals. They predicted that vaccine critical people would be reluctant to engage in interviews because of this atmosphere. Such concerns were also echoed in the team’s fieldwork diaries, which included reference to participants’ worries that, owing to the COVID-19 pandemic context, the research team might be particularly judgemental, seek to change views about vaccination through the research, or that the team might be connected to health authorities.

Given these recruitment challenges experienced, the team decided to use an online recruitment platform, Prolific [14]. Whilst online platforms have limitations such as differences in demographic and attitudinal distributions between online and general populations (see [18], [16] for discussions of some of these issues), the platform possessed advantages for this project. Prolific.com has a range of pre-set screening criteria for sampling of specific populations. The following screening question about scheduled child immunisations was used:

*‘Please rate on a scale from 1 to 7 where 1 is TOTALLY DISAGREEE and 7 is TOTALLY AGREE. “I believe that scheduled immunisations are safe for children”.’*

Given the complex nature of vaccine hesitancy outlined above, this question represents only a rough proxy of the concept. Nevertheless, the team were able to successfully recruit parents of children who self-identified as having some concern about child immunisations, and who lived in the target region.

However, a second methodological problem associated with the COVID-19 pandemic emerged during the interviews and subsequent analysis. Specifically, it became clear that the pandemic, and the fact that COVID-19 vaccines were offered to children as young as 5 in England from April 2022, had shaped how some participants approached the screening question. Whilst the question set by Prolific referred to ‘scheduled immunisations’, and the carefully worded project description clearly referred to ‘childhood vaccination’, it emerged during the interviews that a minority of participants were primarily concerned about COVID-19 vaccination, rather than the recommended childhood schedule. This factor also influenced the way the actual interviews progressed: Our semi-structured interview agenda focussed on questions about childhood vaccines, with only a brief prompt towards the end about whether COVID-19 had influenced their approach towards ‘vaccination in general’. However, in practice, participants found it difficult to distinguish, mentioning the COVID-19 context throughout.

## Interviewing professionals in a challenging healthcare environment

Burnout and staff wellbeing in UK primary care was a pressing issue prior to the COVID-19 pandemic [7]), exacerbated by contextual factors such as the impact of Brexit on staff and staffing [13]. The demands of the pandemic increased stress and fatigue among primary care staff that the researchers were aiming to recruit, with additional pressures including redeployment, inadequate rest, and concerns over provision of personal protective equipment being evidenced in the literature in association with this additional burnout [5], [10]. In this regard, the COVID-19 context also had a detrimental impact on the ability of the social science research team to recruit healthcare professionals as participants.

As with the phase involving parents, several recruitment methods were attempted. These included indirect methods, such as utilising a press release, social media channels, and local radio and TV interviews, as well as more direct methods including personal and institutional networks, direct and gatekeeper contacts, and snowball sampling. Overall, these attempts were only partially successful. Indeed, some of those that did agree to participate used the interview as an opportunity to predict that high stress levels and poor retention exacerbated by the pandemic may hamper further recruitment. For example, one health visitor explained that their local children’s healthcare service was very short staffed and suffered high turnover during the pandemic. When the interviewer asked about snowball sampling, the health visitor replied:

*“I’ll be honest with you, a lot of our health visitors that are trained, not long after training they leave so we’re really struggling to keep them on.”*

Other communications with gatekeepers among general practice nurses, health visitors, and midwives regarding participant recruitment referenced ‘enormous challenges’ being experienced by the health visiting service due to being ‘decimated with public health cuts’, poor retention in general practice nursing, and minimal to no time or energy for research participation outside of their essential work commitments.

## Conclusion

The COVID-19 pandemic has reshaped the possibilities and practicalities of social research. In our study, this context impeded the team’s ability to recruit parent participants, influenced decisions on recruitment methods, and also impacted upon the kinds of vaccine attitudes among participants ultimately recruited. Taken together, this experience added a layer of difficulty in how data analysis was approached. Overall, our main claim is that the pandemic context influenced our ability to investigate and analyse issues around non-COVID-19 vaccination. Some of these issues were made explicit during study recruitment, as the researchers received feedback from those who did and did not agree to be interviewed. Other issues were less explicit, such as the assumptions made about the parameters some parents used to interpret a screening question about childhood immunisation. The experiences described in this short communication have focused on a small regional qualitative study of stakeholder populations, conveying the challenges encountered in carrying out empirical research in the UK under the long shadow of COVID-19. Nevertheless, we can identify some wider recommendations for the use of social research methods to understand vaccine hesitancy beyond the topic of COVID-19 vaccination.

In terms of study recruitment, researchers may historically have assumed a positive but perhaps noisy correlation between general orientations towards vaccination and specific vaccines, or assumed that reference to ‘childhood vaccines’ is self-explanatory, at least in a country such as the UK with a well-publicised schedule. However, based on our research, our first recommendation is that qualitative researchers should take additional care in research planning, and think through the ways in which the experience of the COVID-19 pandemic may continue to influence public discourse and create more varied forms of contemporary vaccine hesitancy.

In practice, for qualitative researchers, this may require modification to recruitment materials, interview/focus group schedules, and approaches to analysis when seeking to carry out research on non-COVID-19 topics. They may also need to leave space for parents to articulate how they see distinctions or similarities between COVID-19-specific and other vaccines. Our argument also matters for quantitative researchers such as those using questionnaire-based methods, who may be subject to new challenges regarding comprehension, recall, and judgement for respondents [4]. In this sense, where resources permit, social researchers interested in vaccination would be well-advised to increase their levels of piloting and pre-testing to understand how the *meta*-context of the pandemic continues to impact their specific area of interest, and adjust methodological choices accordingly.

Existing literature on topics beyond vaccination confirms that recruitment of healthcare professionals for studies can be challenging (see for example [3], [11]). While not created by the COVID-19 pandemic, the pandemic exacerbated these pressures, thus impacting on the longer-term feasibility of social research into vaccination with healthcare professionals. Social researchers aiming to include healthcare professionals in their investigations of vaccination and vaccine hesitancy should therefore ensure there are multiple choices of mode for carrying out interviews, and build in a significant timescale to recruit participants.

Our third recommendation is directed at policymakers. The pandemic has further muddied the boundaries between categories of vaccines. Given COVID-19 vaccines are now available for children in many countries, we suggest that common phrases such as ‘recommended childhood vaccination schedule’, sometimes used by public health authorities, should be rethought. For example, health promoters could adopt phrases such as ‘recommended childhood vaccination schedule and COVID-19 vaccination’ or ‘licensed childhood vaccinations’.

Finally, we would also recommend that more research funding and attention is devoted to exploring the wider social issues that provide the context for vaccine hesitancy. Understanding how vaccine hesitancy is sustained in society is a significant challenge, and whilst crises and controversies are problematic for public health, they also provide opportunities for social researchers. For example, the MMR debate stemming from the controversy over Andrew Wakefield in the UK understandably encouraged more national and international social scientific research on the topic of vaccine hesitancy. However, it has also been argued that we should see such examples as an opportunity to explore wider topical issues, including the complicated nature of public trust in medical authorities and scientific expertise [6]. In the same vein, two decades on from the Wakefield controversy, the COVID-19 pandemic may represent another key historical moment; COVID-19 may have (again) significantly shifted or reframed the relationship between society and vaccines. Social scientific research will play a key role in this ongoing discussion. Indeed, as many regions have moved beyond the emergency phase of the pandemic, social researchers will need to be conscious, not only of whether and how we are witnessing shifts in how vaccine hesitancy is articulated, but also of what this means for how social research is designed, carried out, and analysed.

**Funding**.

This project has received funding from the European Union’s Horizon 2020 Research and Innovation Programme under grant agreement No 965280.

**Ethical approval**.

The collection of interview data as part of this study was approved by the University of Nottingham School of Sociology and Social Policy Research Ethics Committee on 1st December 2021 (Ref: 2122-13-Staff).

## CRediT authorship contribution statement

**Alistair Anderson:** Conceptualization, Data curation, Formal analysis, Investigation, Methodology, Resources, Visualization, Writing – original draft, Writing – review & editing. **Tom Douglass:** Data curation, Investigation, Methodology, Resources, Visualization, Writing – original draft, Writing – review & editing. **Pru Hobson-West:** Conceptualization, Formal analysis, Funding acquisition, Methodology, Project administration, Resources, Supervision, Visualization, Writing – original draft, Writing – review & editing.

## Declaration of competing interest

The authors declare that they have no known competing financial interests or personal relationships that could have appeared to influence the work reported in this paper.

## Data Availability

Data will be made available on request.
